# Disulfide Scrambling in Superoxide Dismutase 1 Reduces Its Cytotoxic Effect in Cultured Cells and Promotes Protein Aggregation

**DOI:** 10.1371/journal.pone.0078060

**Published:** 2013-10-15

**Authors:** Lina Leinartaitė, Ann-Sofi Johansson

**Affiliations:** Department of Biochemistry and Biophysics, Stockholm University, Stockholm, Sweden; National Center of Neurology and Psychiatry, Japan

## Abstract

Mutations in the gene coding for superoxide dismutase 1 (SOD1) are associated with familiar forms of the neurodegenerative disease amyotrophic lateral sclerosis (ALS). These mutations are believed to result in a “gain of toxic function”, leading to neuronal degeneration. The exact mechanism is still unknown, but misfolding/aggregation events are generally acknowledged as important pathological events in this process. Recently, we observed that demetallated apoSOD1, with cysteine 6 and 111 substituted for alanine, is toxic to cultured neuroblastoma cells. This toxicity depended on an intact, high affinity Zn^2+^ site. It was therefor contradictory to discover that wild-type apoSOD1 was not toxic, despite of its high affinity for Zn^2+^. This inconsistency was hypothesized to originate from erroneous disulfide formation involving C6 and C111. Using high resolution non-reducing SDS-PAGE, we have in this study demonstrated that the inability of wild-type apoSOD1 to cause cell death stems from formation of non-native intra-molecular disulfides. Moreover, monomeric apoSOD1 variants capable of such disulfide scrambling aggregated into ThT positive oligomers under physiological conditions without agitation. The oligomers were stabilized by inter-molecular disulfides and morphologically resembled what has in other neurodegenerative diseases been termed protofibrils. Disulfide scrambling thus appears to be an important event for misfolding and aggregation of SOD1, but may also be significant for protein function involving cysteines, e.g. mitochondrial import and copper loading.

## Introduction

 Mutations in the superoxide dismutase 1 (SOD1) gene account for more than 20 % of the familial cases of amyotrophic lateral sclerosis (ALS), an aggressive neurodegenerative disorder characterized by degeneration of upper and lower motor neurons. SOD1 is a homodimeric enzyme where the monomeric 153-residue subunit is folded into a β-barrel conformation connected by seven loops. Loop IV, coordinating one Zn^2+^ ion, is attached to strand 8 in the β-barrel via a disulfide bond formed by C57 and C146. Together with loop VII, loop IV creates a channel guiding superoxide to the active site where Cu^2+^ is essential for the biological function of SOD1 as a superoxide scavenger. 

 The lack of motor neuron disease in SOD1 knock-out mice [1] and the normal enzymatic activity conferred by some of the SOD1 mutants [2,3] suggest a cytotoxic gain of function similar to the disease causing proteins in other neurodegenerative disorders like Alzheimer’s disease and Parkinson’s disease. One hypothesis for the neuronal toxicity caused by mutant SOD1 is the formation of protein aggregates. In support of this idea, intracellular protein inclusions containing SOD1 have been identified in transgenic mice expressing human mutant SOD1 [4], as well as in familial ALS with SOD1 mutations [5] and in sporadic ALS [6]. In addition, over-expression of mutant SOD1 in cultured cells *in vitro* results in aggregation and neurotoxicity linked to inter-molecular disulfide cross-linking of the free cysteines C6 and C111 [7,8].

 The cell culture system used in the study presented herein differs however significantly from these transfection-based systems as recombinantly produced SOD1 is added directly to the cell culture media of neuronal cells. Demetallated apoSOD1 with both C6 and C111 substituted for alanine has previously been observed to exert toxicity in this system in its native folded form, i.e. non-aggregated. Interestingly, destabilization, metallation, or mutations obstructing the Zn^2+^ affinity of the protein all results in a non-toxic protein [9]. The toxic effect was therefore concluded to result from Zn^2+^ chelation, depriving the cells of this essential metal. The relevance of this cell model for ALS is discussed in the original paper by Johansson et al. [9]. For the purpose of the study presented in this paper, the cell viability assay is better perceived as a Zn^2+^ binding assay where toxic SOD1 proteins represent native, folded SOD1 variants with high affinity for Zn^2+^. 

 The findings presented in Johansson et al. are herein extended by data providing evidence for non-native intra-molecular cysteine bond formation in wild-type apoSOD1 which renders the protein non-toxic, i.e. Zn^2+^ deficient. The data is consistent with a model where C6 initially attacks the native disulfide, forming a non-native disulfide bond, which in turn is attacked by C111 creating an additional non-native disulfide. Moreover, the monomeric apoSOD1 variants capable of forming non-native disulfides in cell culture media were observed to aggregate into Thioflavin T-positive oligomers under physiological conditions without agitation.

## Results

### Wild-type apoSOD1 is non-toxic to cultured neuroblastoma cells but regain toxicity by cysteine alkylation

**Figure 1:**
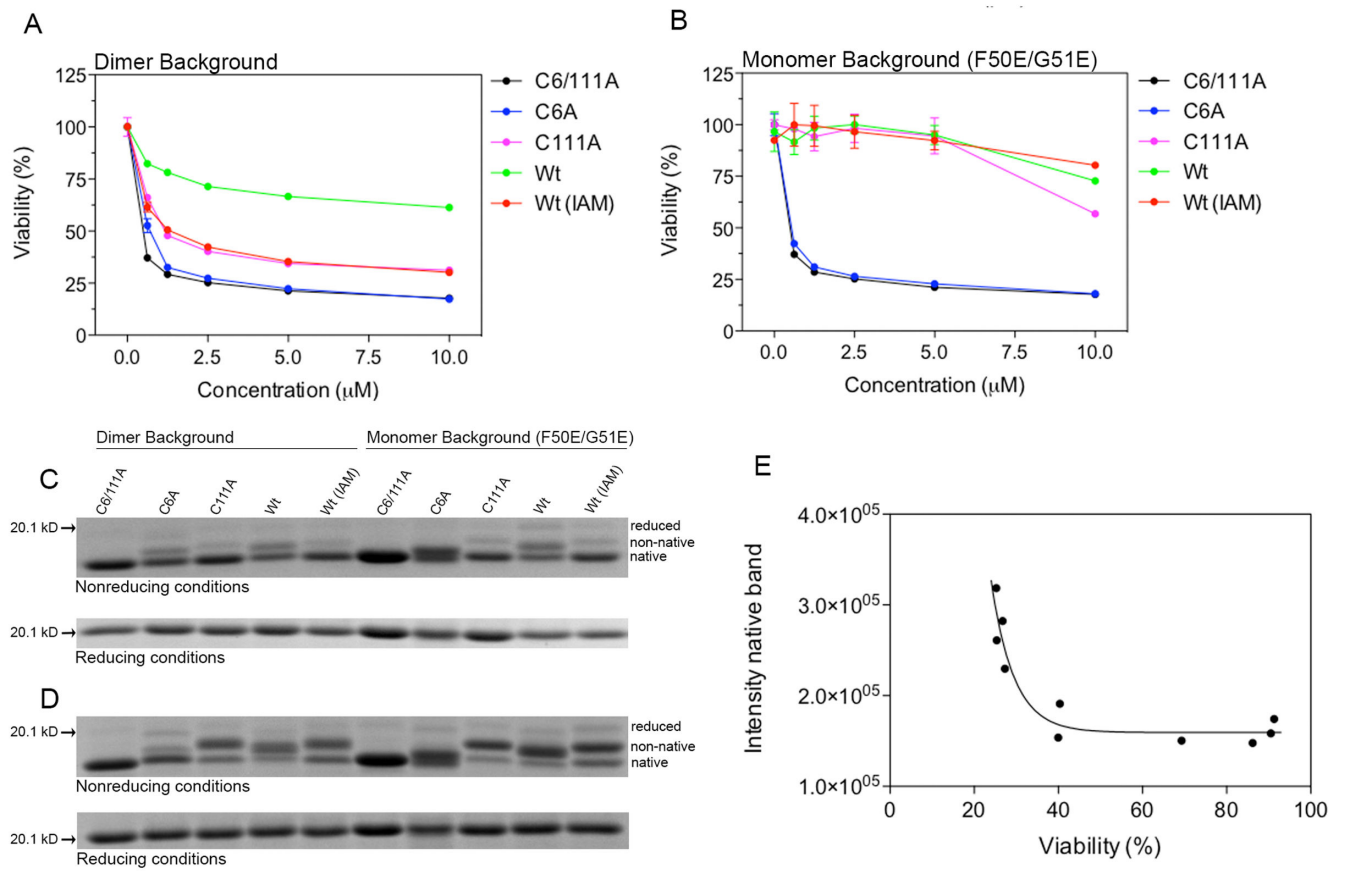
ApoSOD1 variants displaying reduced cytotoxicity migrate slower in non-reducing SDS-PAGE. ApoSOD1 in dimeric or monomeric form were added to the cell media of cultured SH-SY5Y cells and incubated for 72 h. Proteins were added in duplicate wells and cell viability was measured with the MTT assay. Cell viability is presented as the mean percentage viability of the buffer control ± range. (**A**) Dimeric wild-type apoSOD1 induced very little toxicity, whereas cysteine alkylation with iodoacetamide (IAM) or substitution of C111 for alanine restored toxicity almost, but not completely, to the level of apoSOD1 C6/111A. (**B**) Only monomeric apoSOD1 with C6 or both C6 and C111 substituted for alanine were toxic, whereas wild-type, wild-type (IAM) and C111A were essentially non-toxic. (**C**) Non-reducing and reducing SDS-PAGE of apoSOD1 samples before adding them to the cell culture. All proteins mainly migrate as native SOD1 (compare with C6/111A). (**D**) Non-reducing and reducing SDS-PAGE of apoSOD1 samples collected from the cell culture after 72 h of incubation. ApoSOD1 variants displaying reduced toxicity all migrate with a slower rate under non-reducing conditions compared to the toxic proteins C6/111A and C6A. Under reducing conditions, all proteins migrate with the same rate. (**E**) The band intensity of the native band was clearly correlated to cell viability. The cellular response for each apoSOD1 variant was determined by the area under the viability curve in A and B.

 The initial observation leading to this study was the low toxicity of dimeric wild-type apoSOD1 (Figure 1A), despite of being presumably native with high affinity for Zn^2+^. We reasoned that the oxidative environment of the cell culture media [10] could promote cysteine cross-linking, which could potentially disturb the Zn^2+^ affinity of the protein. In an attempt to prevent this, dimeric wild-type holoSOD1 was modified with iodoacetamide (IAM) to alkylate free cysteines. The alkylated protein was subsequently demetallated and added to the cell media of cultured SH-SY5Y neuroblastoma cells [11]. In favor of the disulfide cross-linking hypothesis, cysteine-alkylation did increase the toxicity of wild-type apoSOD1 significantly (Figure 1A). To further evaluate the individual effect of the free cysteines C6 and C111, these were selectively substituted for alanine. Interestingly, the level of cell toxicity of apoSOD1 C111A was virtually identical to the alkylated wild-type protein (Figure 1A), suggesting that primarily C111 is modified in the alkylation procedure. ApoSOD1 C6A was more toxic to the cells compared to C111A and reduced cell viability almost to the level of C6/111A (Figure 1A), indicating an essential role for C6 in non-native disulfide formation. 

 To examine the effects of the same mutations in monomeric SOD1, the F50E/G51E double mutation was introduced [12] creating a monomeric protein unable to form native dimers. In agreement with the results for dimeric apoSOD1, monomeric wild-type apoSOD1 was non-toxic to the cells whereas substituting C6 and C111 for alanine creates a toxic protein as previously observed [9] (Figure 1B). Like the dimeric protein, also monomeric apoSOD1 C6A exert a level of toxicity similar to the C6/111A protein (Figure 1B). In contrast to the dimeric protein, however, alkylated monomeric wild-type apoSOD1 did not differ in cell viability from the monomeric un-modified wild-type protein; both proteins were non-toxic to the cells (Figure 1B). Nevertheless, this agrees with the notion of C111 being modified by the alkylation procedure, as the monomeric C111A protein also was essentially non-toxic even though cell viability is somewhat reduced for the highest concentration. 

### Iodoacetamide alkylates a single cysteine in metallated wild-type SOD1

 To evaluate the efficiency of the alkylation procedure, both monomeric and dimeric wild-type SOD1 subjected to cysteine alkylation were analyzed with MALDI-TOF MS to determine the number of alkylated cysteines in the proteins. An alkylated cysteine increases the mass with 57 Da. Both proteins mainly displayed a peak corresponding to one cysteine being modified, for the dimer the 15860.5 Da peak (Figure S1A) and for the monomeric protein the 15914 Da peak (Figure S1B). A mass of 15917 corresponding to dimeric SOD1 with two alkylated cysteines was also detected but with much lower intensity. Consistently, a mass of 15971 representing monomeric SOD1 with two alkylated cysteines was barely detected. Mass numbers representing three or four alkylated cysteines could not be identified for either dimeric or monomeric SOD1. Even though the MS data don’t reveal the identity of the cysteine being alkylated, it clearly shows that primarily one cysteine per monomer is modified. Taken together with cell viability data, however, C111 stands out as the main candidate for modification (Figure 1A&B), which is not unexpected since C111 is known to be surface exposed in the metallated protein. C6, on the contrary, is buried in metallated holoSOD1 but become highly solvent accessible in apoSOD1 [13]. Note that the alkylation procedure is performed on the holo protein, before apo treatment (see experimental procedures).

### High cell viability coincide with the presence of a shifted band in non-reducing SDS-PAGE representing SOD1 with a non-native intra-molecular cysteine bond

 To determine the cysteine cross-linking status of apoSOD1, all proteins were separated on a SDS-PAGE gel under reducing and non-reducing conditions, both before (Figure 1C) and after incubation with the cells (Figure 1D). Since no serum proteins were present in the samples collected from the culture media, gels were stained with coomassie blue.

 Reduced monomeric and dimeric apoSOD1 C6/111A (MW: 15 kDa) both migrated with the same rate as the 20.1 kD molecular weight marker, whereas the non-reduced protein migrated more rapidly (Figure 1C&D). The difference in migration rate for reduced and non-reduced SOD1 have previously been observed by others [14,15]. The C6/111A band on the non-reducing gel presumably represents the native protein with the native C57-C146 disulfide intact, as no erroneous cross-linking involving C6 and C111 can occur. 

 When analyzing the proteins before applying them to the cell culture, all proteins mainly migrated as the native protein band on the non-reducing gel (Figure 1C) with the exception of C6A and wild-type where weak bands of slower migrating species are observed. This suggests that all proteins contain primarily the native C57-C146 cysteine bond when adding them to the cells with little presence of non-native or higher molecular weight cross-linked species (Figure S2). This conclusion was collaborated by melting curves for dimeric and monomeric apoSOD1 C6/111A, wild-type and wild-type (IAM), displaying nice transitions curves indicative of a native protein (Figure S3). 

 For proteins collected from the cell culture after 72 h of incubation, both dimeric and monomeric C6A migrated primarily with the same rate as C6/111A (Figure 1D), although a week band with a slower migration rate was observed for dimeric C6A. This band, however, was present already at the initiation of the experiment and did not increase over time. The gel pattern for dimeric and monomeric C6A is thus in agreement with their similar effect on cell viability compared to C6/111A and also demonstrates that these proteins were not compromised during the duration of the cell experiment. 

 In contrast to the C6A protein, the migration rate of C111A, wild-type and wild-type (IAM) in both dimeric and monomeric forms were clearly slower compared to C6/111A with its native disulfide intact but noticeably more rapid than the reduced protein (Figure 1D). The placement of this band in-between reduced and non-reduced apoSOD1 is consistent with the formation of a non-native intra-molecular disulfide bond, in contrast to inter-molecular cross-linking, as the protein mainly migrates as a monomeric protein in SDS-PAGE but just slightly shifted compared to the native protein band. Interestingly, both dimeric and monomeric wild-type migrates slightly faster than C111A and wild-type (IAM) but still more slowly than native SOD1. This observation could indicate that presence of cysteine in position 111 leads to formation of a different non-native cysteine bond compared to SOD1 variants containing only C6. Higher molecular weight cysteine cross-linked species are detected but at much lower intensity (Figure S4). Under reducing conditions, all proteins migrated with the same rate. Note the virtually identical gel pattern for the dimeric and monomeric wild-type (IAM) and C111A proteins (Figure 1D), again collaborating the conclusion of C111 being modified by the alkylation procedure. 

 To highlight the connection between low cell viability and presence of native protein the intensity of the native protein band was quantified and correlated to cell viability (Figure 1E), displaying a clear correlation between low cell viability and intensity of native apoSOD1. Correspondingly, the intensity of the non-native species correlates with high cell viability (data not shown). 

 In summary, the data from the SDS-PAGE analysis is consistent with the formation of a non-native intra-molecular disulfide bond. The free cysteine involved in this non-native disulfide appears to be predominantly C6, as substituting C6 for alanine reverts both dimeric and monomeric SOD1 back to a toxic protein migrating with the same rate as the native protein in SDS-PAGE. Furthermore, the high cell viability associated with this non-native species suggests a compromised Zn^2+^-site. 

### The ability of monomeric apoSOD1 to form a non-native intra-molecular cysteine bond coincides with high aggregation potential

 To evaluate the aggregation potential of the different SOD1 variants, all monomeric and dimeric apoSOD1 proteins were incubated in 96 well plates at 37°C for a duration of 83 h (5000 min) together with Thioflavin T (ThT), a well-known beta-sheet binding fluorescent dye commonly used to study protein aggregation [16]. The proteins were diluted in PBS pH 7.4, resembling the ionic strength and pH of the cell culture media. No agitation was applied. 

**Figure 2:**
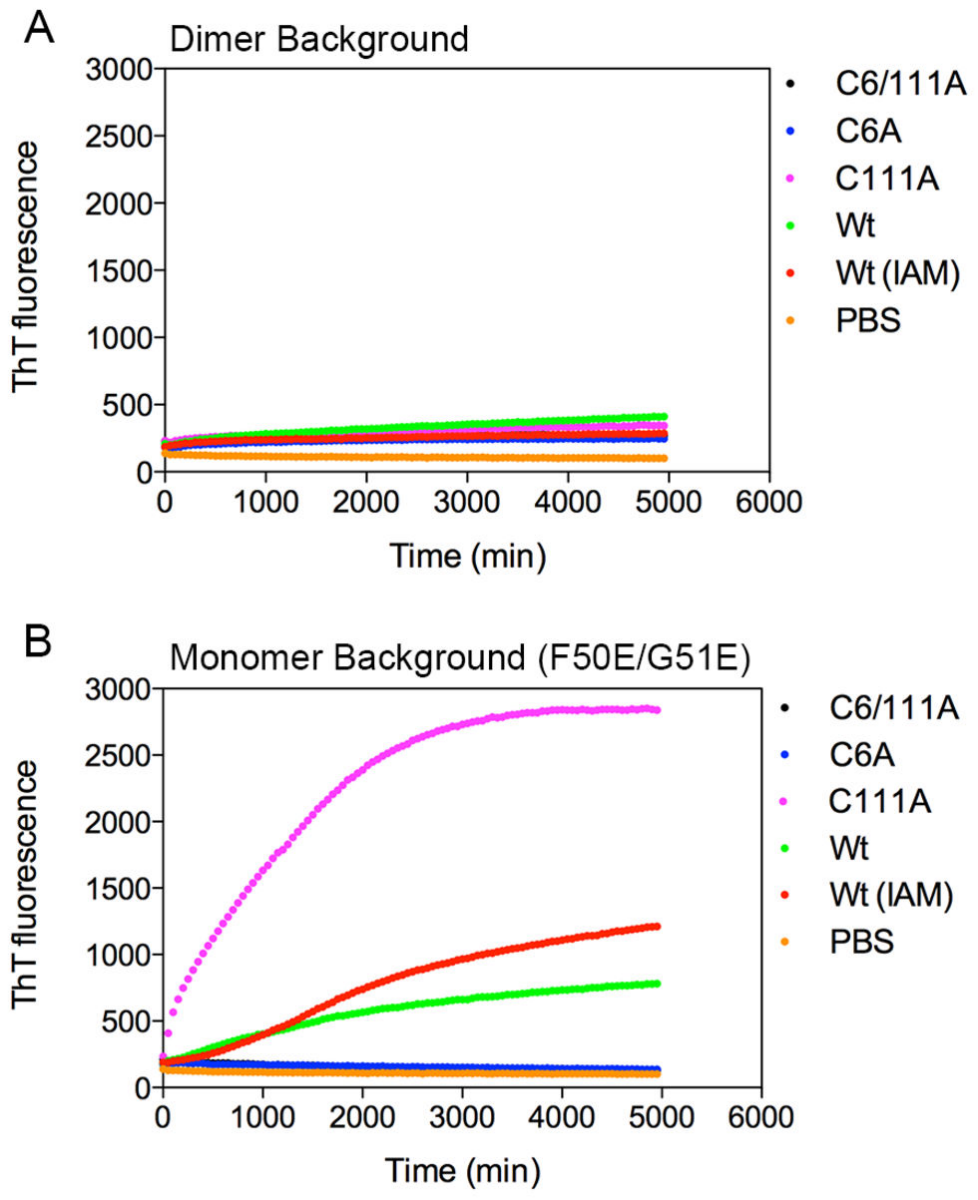
Monomeric SOD1 variants with slow migration rate in non-reducing SDS-PAGE aggregate into ThT positive aggregates. ApoSOD1 (90 μM) was aggregated with 10 μM ThT in PBS without agitation for 83h (4950 min). Data points were plotted with 50 min interval. Each data point represents the mean of three separate experiments run in duplicate. Individual aggregation curves are presented in Figure S5. (**A**) None of the dimeric proteins aggregate under these conditions. (**B**) Monomeric C111A display a high ThT signal. Also wild-type and wild-type (IAM) aggregate into ThT positive aggregates, with a somewhat higher signal for wild-type (IAM).

 Demetallated monomeric wild-type, C111A and wild-type (IAM) all aggregated (Figure 2B), which are the same proteins displaying full cell viability and migration rate consistent with a non-native intra-molecular cysteine bond in non-reducing SDS-PAGE. This identifies monomeric apoSOD1 with scrambled disulfides as a possible precursor for SOD1 aggregation under physiological conditions. None of the dimeric proteins displayed a high ThT signal, demonstrating that dimeric apoSOD1was unable to form ThT positive aggregates in PBS (Figure 2A) despite of the fact that dimeric wild-type, C111A and wild-type (IAM) all were capable of forming non-native intra-molecular disulfide bonds in culture media (Figure 1D). All individual aggregation curves for the separate experiments are included in Figure S5, displaying the spread of the data. 

### Inter-molecular cysteine cross-linking stabilizes soluble SOD1 oligomers

**Figure 3:**
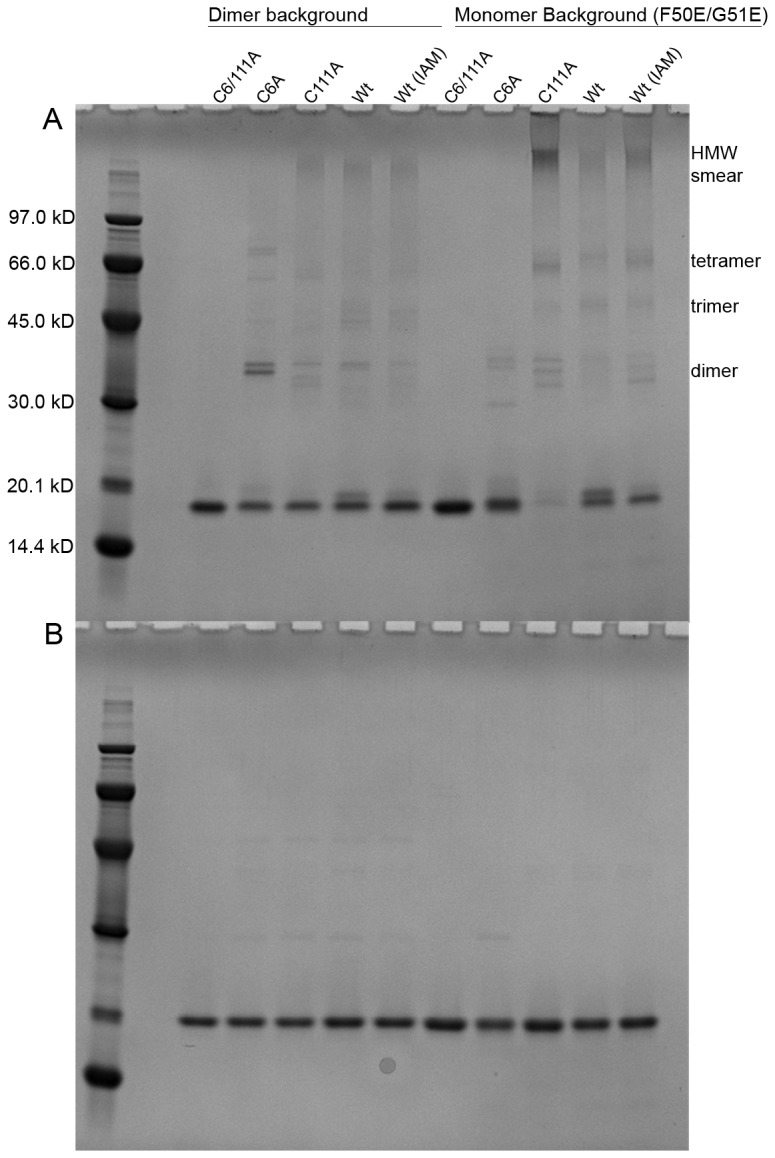
SOD1 oligomers are stabilized by intermolecular disulfide formation. (**A**) Non-reducing and (**B**) reducing SDS-PAGE of aggregated apoSOD1 collected after 83 h of incubation in PBS without agitation. Monomeric apoSOD1 wild-type, C111A and wild-type (IAM) all form both low molecular weight and high molecular weight species stabilized by inter-molecular disulfide cross-linking as demonstrated by their disappearance in the reducing gel. All dimeric apoSOD1 variants mainly migrate as native monomer on the non-reducing gel with only very weak staining for higher molecular weight species.

 To further evaluate the nature of the SOD1 aggregates formed in the aggregation experiment, all SOD1 variants were collected at the endpoint of the incubation, centrifuged and analyzed with non-reducing SDS-PAGE. The most aggregation prone SOD1 variant, monomeric C111A, contained a high molecular weight smear and some lower molecular weight species consistent with dimers, trimers and tetramers (Figure 3A). Under reducing conditions, all oligomeric species migrated as native SOD1 monomer (Figure 3B) revealing cysteine cross-linking as an important factor for stabilization of these aggregates. The equal intensity of all proteins under reducing conditions determines the high solubility of these aggregates as they are not pelleted at 13 300 x g. 

 The less aggregation prone monomeric wild-type and wild-type (IAM) variants displayed similar gel profiles compared to C111A but with additional monomeric protein remaining (Figure 3A). All dimeric SOD1 proteins contained a significant amount of native monomer after the full duration of the incubation consistent with their low aggregation propensity.

### Electron micrographs of ThT positive aggregates formed from monomeric apoSOD1 contain small curvilinear oligomeric species

**Figure 4:**
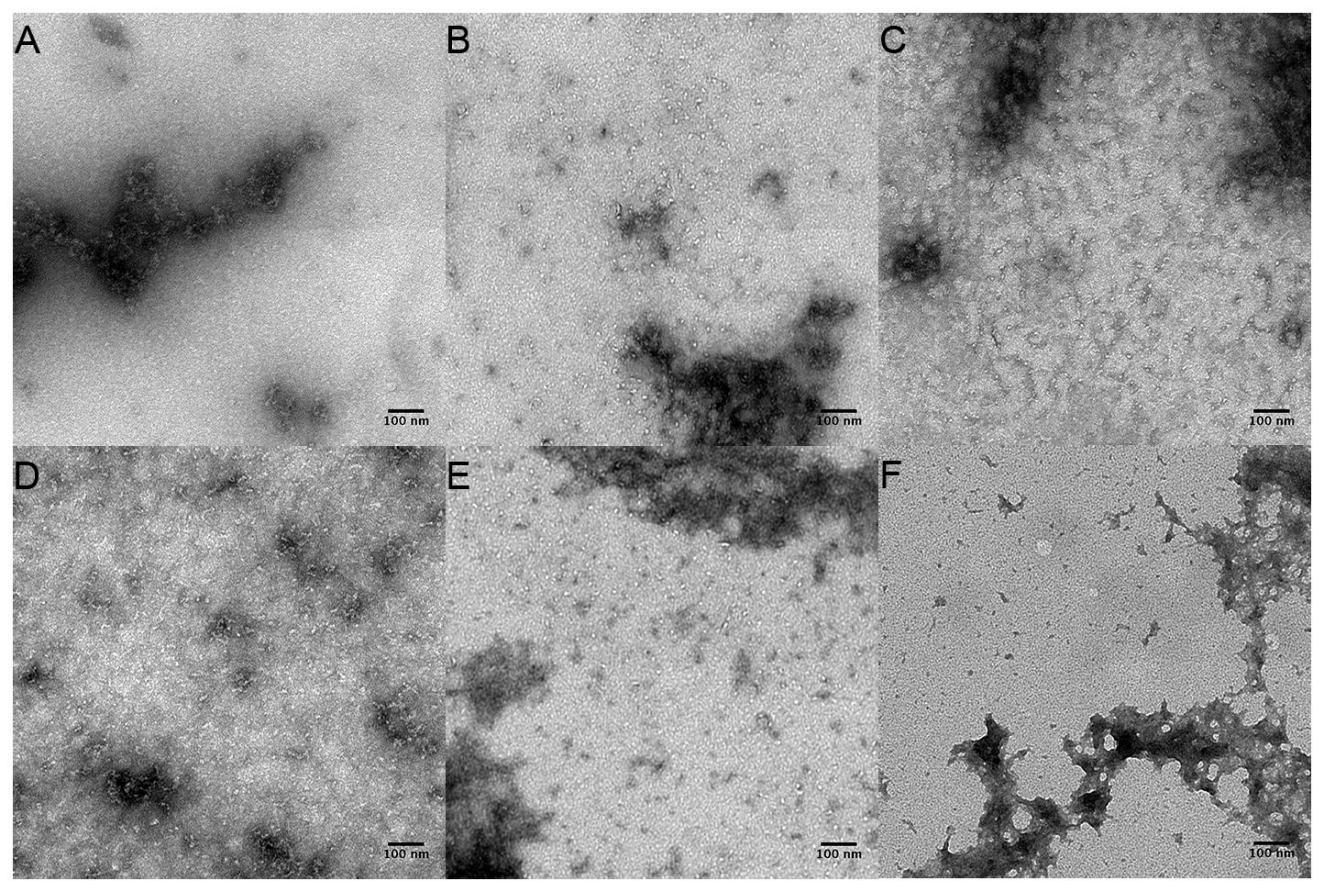
Electron micrographs of aggregated monomeric apoSOD1. Aggregated monomeric apoSOD1 (on a F50E/G51E background) was applied to carbon-coated copper grids and stained with uranyl acetate. (**A**) wild-type (**B**) C6A (**C**) C111A (**D**) wild-type (IAM) (**E**) C6/111A (**F**) negative control (PBS). Monomeric apoSOD1 C111A and wild-type (IAM) contained a large amount of distinct curvilinear oligomers, whereas the aggregates formed from apoSOD1 wild-type were more “fuzzy” and clustered together. Small oligomers in less abundance were observed for apoSOD1 C6A and C6/111A.

 To visualize the ThT positive protein aggregates formed from monomeric apoSOD1, all monomeric SOD1 variants were collected from the 96 well plate and diluted in H_2_O to 25 μM. The samples were applied to carbon coated formvar grids, stained with uranyl acetate and examined in an electron microscope. Monomeric demetallated wild-type (Figure 4A), wild-type (IAM) (Figure 4D) and C111A (Figure 4C) all formed curvilinear aggregates resembling protofibrils described for several other proteins and peptides involved in neurodegenerative disorders [17-20]. This curvilinear appearance was especially clear for the aggregates formed from C111A and alkylated wild-type protein (Figure 4C&D). The monomeric wild-type protein sample appeared somewhat different than C111A and wild-type (IAM) with small curvilinear oligomers coalescing into larger fuzzy aggregates (Figure 4A). This observation, however, agrees with the faster migration of monomeric wild-type in non-reducing SDS-PAGE compared to C111A and alkylated SOD1 (Figure 1D), indicating that this band represents an alternative non-native species forming aggregates with somewhat different properties/morphology. Electron micrographs of monomeric C6A (Figure 4B) and C6/111A (Figure 4E) appeared to contain some aggregates, though smaller and in less abundance, in agreement with the low ThT signal for these proteins.

 As a negative control, PBS was applied and stained in the same manner. Although some artifacts from the staining procedure were observed (Figure 4F), these are clearly distinguishable from the aggregates in the protein containing samples. As an extra control the C111A protein was also stained with PTA, a stain not associated with the same artifacts as the combination of uranyl acetate and PBS. Also in this case the same type of oligomers was observed (Figure S6), ranging in diameter from 3-7 nm and with a length of up to 200 nm. 

## Discussion

### Disulfide scrambling in dimeric apoSOD1

 The data presented herein indicate a crucial role for C6 in the formation of non-native intra-molecular disulfide bonds in apoSOD1. Even though C6 in holoSOD1 is buried within the core of the β-barrel, it becomes highly solvent accessible upon demetallation [13]. The proximity of C146 to C6 implicates C146 as the most likely candidate for nucleophilic attack by the C6 thiolate (1), even though an interaction with C57 cannot be excluded. A direct attack of C111 on the native C57-C146 disulfide is contradicted by the identical Zn^2+^ chelating capacity and gel-migration rate of C6A compared to C6/111A. However, C111 still appears to have a significant role in disulfide scrambling, as the dimeric C111A protein is a considerably better Zn^2+^ chelator than wild-type SOD1. 

 These observations can be reconciled into a model where C111 attacks the non-native C6-C146 disulfide only, but not the native C57-C146 disulfide bond, creating an additional non-native disulfide formed by C146 and C111 (2). 

(1) C6+C57 − C146→C6 − C146+C57

(2) C6 − C146+C111→C111 − C146+C6

**Figure 5:**
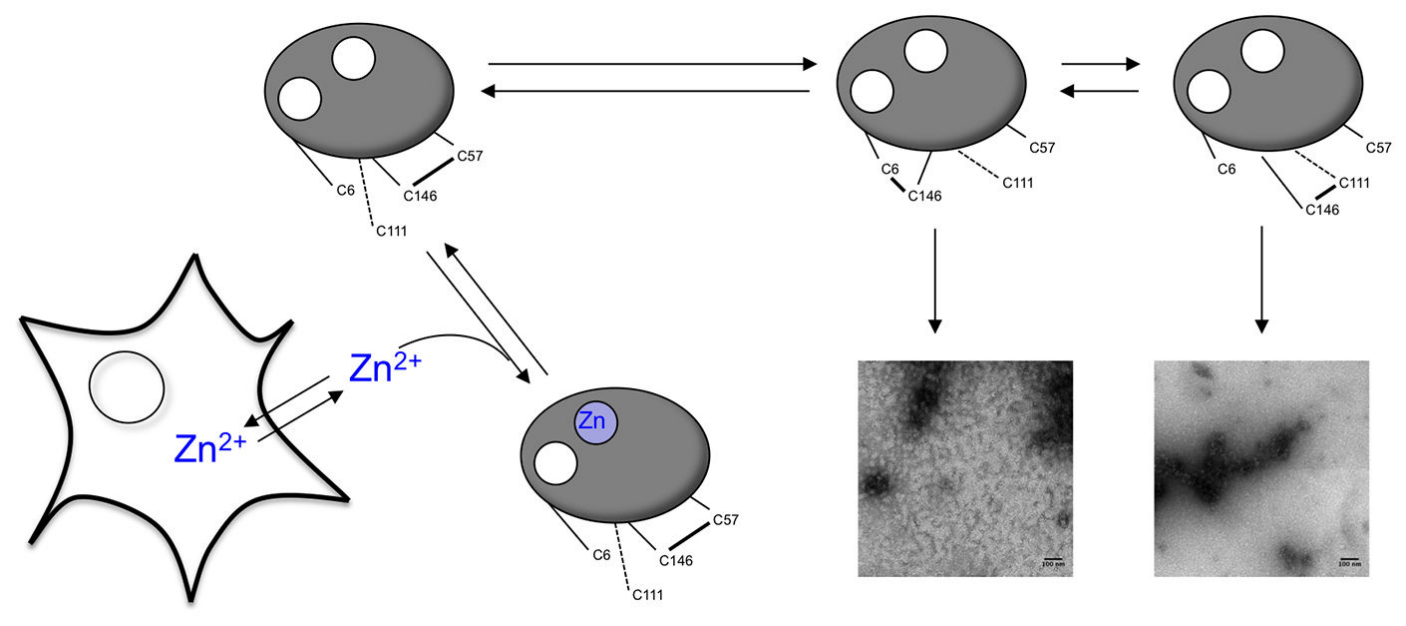
Schematic representation of the effect of disulfide scrambling on the Zn^2+^ chelating capacity and aggregation of apoSOD1. ApoSOD1 chelate Zn^2+^ from the culture media, thereby draining the cells of this essential metal and reducing cell viability. Formation of non-native disulfide bonds between C6/C111 and C146 (alt. C57) reduce the Zn^2+^-chelating capacity of apoSOD1 and sets the protein up for aggregation into soluble oligomers stabilized by inter-molecular disulfides.

The dimeric wild-type protein would thus according to this model ultimately acquire the C111-C146 bond, whereas the C111A and C111 (IAM) proteins would retain the C6-C146 bond (Figure 5). This agrees with the difference in Zn^2+^ chelating capacity for these proteins and their slightly different migration rate in non-reducing SDS-PAGE as well as different appearance in electron microscopy. The presence of a weak band in C6A with similar migration rate to the non-native wild-type band suggests that a small fraction of this protein has acquired the C111-C146 bond in absence of C6. This observation does not necessarily contradict the proposed model as the C111-C146 bond could have formed during the apo procedure, where the protein is kept in an unfolded state for an extended time period. In support of this, the intensity of the non-native C6A band does not increase over the time-course of the experiment (compare Figure 1C and 1D) indicating that the C6A mutation prevents formation of the C111-C146 bond in folded apoSOD1. 

 Reduced Zn^2+^ affinity as a consequence of non-native intra-molecular disulfide formation is not surprising as loop IV containing the Zn^2+^ binding residues is anchored to the β-barrel by the C57-C146 cysteine bond. Disruption of the native disulfide due to disulfide scrambling is thus likely to compromise the Zn^2+^ affinity of the protein. This is collaborated by the low toxicity of apoSOD1 lacking all four cysteines [9] as well as the diminished affinity of reduced wild-type SOD1 for Zn^2+^ [21]. However, even though cell toxicity in this cell model is strongly associated with the Zn^2+^- binding capacity of apoSOD1, further experiments are of essence to validate and further investigate the Zn^2+^-binding properties of scrambled SOD1. 

 The suggested model for disulfide scrambling in apoSOD1 is interestingly enough very well supported by the mass spectrometry data presented by Toichi et al. where alkylation and tryptic digestion of aggregating apoSOD1 revealed the presence of peptides representing SOD1 with non-native intra-molecular disulfides, including C6-C146 and C111-C146. The peak intensities of the C6-C146 peptides were increased as early as after 2 h of incubation but decreased at 24 h whereas the peak intensities of the C111-C146 peptides were barely detected initially but built up with time, indicating that C6-C146 disulfide formation precedes formation of the C111-C146 disulfide [22]. Moreover, disulfide scrambling enabling formation of the C111-C146 bond has previously been observed for wild-type apoSOD1 [15,23], further supporting this model. 

### Disulfide scrambling in monomeric apoSOD1

 Judging from the SDS-PAGE data alone, monomeric apoSOD1 form the same non-native intra-molecular bonds as dimeric apoSOD1. In spite of this, replacing C111 in the apoSOD1 monomer does not affect the Zn^2+^-chelating capacity to the same extent as in the dimeric protein. The native disulfide in monomeric apoSOD1 is, however, considerably more flexible and prone for attack by neighbouring cysteines because of the protein’s inability to dimerise. Monomerisation thus drives the equilibrium in (1) towards formation of the C6-C146 bond reducing the time frame for Zn^2+^ chelation. Dimerisation on the other hand stabilizes the structural surrounding of the native disulfide as the dimer interface is formed by the disulfide loop and β-strand 8. As a consequence will dimeric SOD1 variants with C6 intact be able to chelate more Zn^2+^ compared to monomeric SOD1 (before the C6-C146 disulfide is formed), thus reducing the viability of the cells.

 The different susceptibility of monomer and dimer to form non-native disulfides is however not apparent in the SDS-PAGE analysis, as the proteins are collected from the highest concentration (10 μM) after the full duration of the cell experiment. Only a small fraction of the protein will coordinate Zn^2+^ as the Zn^2+^ ions provided by the cells is estimated to result in a final concentration of 420 nM in the culture media (based on a Zn^2+^ level of 0.4 mol/cell) [9,24]. This is also apparent from the maximum toxicity response already reached at low μM concentrations. The majority of the protein will thus remain in apoform and eventually form the non-native cysteines bonds in (1) and (2).

### Disulfide scrambled SOD1 as a precursor for aggregation

 A significant fraction of mutant SOD1 from transgenic mice [8,14,25-27], transfected cells [7,8,14,27] and purified SOD1 [25,28] migrates as cysteine cross-linked higher molecular weight species in non-reducing SDS-PAGE. These observations have been explained by inter-molecular disulfide cross-linking promoting aggregation, a conclusion supported by lower accessibility of free cysteines to the solvent during aggregation of wild-type apoSOD1 [29]. However, as inter-molecular disulfide cross-linked aggregates accumulate in the later stages of the disease in transgenic mice [26], these may not be essential for aggregation onset but help to stabilize higher molecular weight aggregates as suggested by Wang et al. [27]. The most immature form of SOD1, i.e. reduced and devoid of metals has instead been put forward as an initial aggregation precursor [26,27,30]. This is mainly based on the high aggregation potential of reduced apoSOD1 *in vitro* [30,31] and further supported by studies in mutant SOD1 mice demonstrating that a significant fraction of aggregated SOD1 migrates as reduced SOD1 in non-reducing SDS-PAGE [15,26]. However, the similar migration rate for reduced SOD1 and SOD1 with non-native disulfides demonstrated in this report raises the possibility that previous studies may have mistaken disulfide scrambled SOD1 for reduced protein. 

 The aggregation data presented herein, i.e. the high ThT signal and the oligomeric structures observed in PBS for monomeric apoSOD1 variants capable of forming non-native disulfides in cell media indicate disulfide scrambled monomeric apoSOD1 as an alternative precursor for SOD1 aggregation. Disulfide scrambling involving C6 thus appears to be a key event for initiation of aggregation, as also proposed by Toichi et al. [22]. Moreover, C6 has been implicated in the formation of soluble disulfide linked high molecular weight oligomers [8], in agreement with the high molecular weight aggregates observed in this study which also were stabilized by inter-molecular disulfides (Figure 3). Disulfide scrambling thus appears to induce structural changes in the apoSOD1 structure, facilitating inter-molecular disulfide formation. This could occur either directly by thiol-disulfide exchange, i.e. the attack on a non-native disulfide by free cysteines in neighboring SOD1 molecules, or oxidation of remaining free cysteines. 

 The inability of dimeric apoSOD1 to form non-native disulfides and aggregate in PBS, despite of its ability to form disulfide-scrambled species in cell media, may indicate the necessity of a second trigger for these events to occur in PBS. Destabilization of the structure induced by monomerisation may be such a trigger, considering that changes in reaction conditions such as pH, heat [32] and chemical denaturation [31,33], as well as ALS-associated SOD1 mutations [32,34] are known to destabilize the SOD1 molecule and induce aggregation.

### The role of C6 and C111 in mitochondrial import

 C6 is a highly conserved residue in the SOD1 amino acid sequence [27] whereas C111 is common to be serine in other species, e.g. in murine SOD1. Conservation of amino acid residues may indicate strong influence on function or folding kinetics [35]. In fact, C6 and the structure surrounding this residue have been observed to be a part of the folding nucleus of SOD1 [36] but no direct effects on protein function has been attributed to this residue. 

 Three familiar ALS mutations have been described in C6; C6F [37], C6G [38] and C6S [39]. C6F and C6G are highly penetrant with a rapid disease progression, whereas C6S display reduced penetrance and long survival time. Both C6F and C6G are however highly insoluble in contrast to the soluble C6S protein [7,14,40], providing an explanation for this discrepancy. The C6S mutation has instead been associated with decreased mitochondrial localization [40], which could explain its pathology.

 Only one familiar ALS mutation in position C111 has been reported, the C111Y mutation associated with variable onset and disease duration [41]. Also the C111Y mutation increases the fraction of insoluble protein [14]. Moreover, C111 is implicated in mitochondrial import as substitution of C111 for serine reduces mitochondrial localization of SOD1 [40], similar to C6S. Both C6 and C111 thus seem to be important for mitochondrial import of SOD1. Interestingly enough, mitochondrial import of SOD1 only occurs in its reduced apo form [42]. Once inside the mitochondria, SOD1 interact with CCS and matures into its fully enzymatically active metallated disulfide intact form [40,42]. 

 An intra-molecular cysteine bond between C6/C111 and C146 could thus be hypothesized to “lock” the SOD1 molecule in a demetallated apo state with C57 reduced, enabling mitochondrial import. In this way, C57 would also be free to form an inter-molecular cysteine bond with C229 in CCS [43] allowing copper loading and full maturation of SOD1. 

## Concluding Remarks

 As SOD1 folds and maturate into an active fully metallated enzyme, a large number of intermediates are populated. It is conceivable that not just one, but several of these can be prone for aggregation. The occurrence of several parallel aggregation routes can explain the variation in onset, duration and phenotype for different familiar ALS mutations in SOD1. The formation of non-native intra-molecular disulfides provides an additional route for aggregation in addition to the ones already described in the literature. Disulfide scrambling may however not always be a harmful event but might also play an important role in folding, maturation and function of SOD1, e.g. mitochondrial import. Familiar ALS mutations or changes in the cellular environment could potentially increase the pool of scrambled SOD1 to a point where a critical concentration is reached, enabling aggregation and pathological events.

## Experimental Procedures

### Protein expression and purification

 SOD1 was co-expressed with the yeast Cu-chaperone CCS in *E.Coli*. The bacterial cultures were grown at 23 °C, and CuSO_4_ (3 mM) and ZnSO_4_ (30 μM) were added upon induction. Purification was done by heat denaturation (55 °C for 30 min) followed by ammonium sulfate precipitation, gel filtration (S100 Sephacryl 100, Amersham Pharmacia) and ion-exchange chromatography (Q-Sepharose) [44]. 

### Cysteine alkylation

 Purified “as it comes” protein in H_2_O was alkylated by addition of 1/10 volume of 150 mM iodoacetamide prepared in either H_2_O or 500 mM Tris-HCl, pH 8.5. The protein solution was incubated for 1 h at RT and dialyzed against water to remove excess iodoacetamide. Alkylated proteins were analysed with MALDI-TOF (Protein Analysis Center (PAC), Karolinska Institute, Stockholm, Sweden).

### Apo procedure

 Apo protein was prepared by adding the protein solution to a Slide-a-Lyzer mini dialysis tube (Pierce) placed in a solution of 6 M of guanidinium chloride, 50 mM of Formate buffer pH 3.5 and 250 mM of EDTA for a minimum of 4 h at room temperature. The protein was subsequently dialyzed against 10 mM MES buffer pH 6.3 for cell viability experiments or PBS pH 7.4 (50 mM phosphate 100 mM NaCl) for aggregation experiments. 

### Cell cultures

 Human neuroblastoma SH-SY5Y cells [11] were cultured in Dulbecco’s Modified Eagle Medium (D-MEM, Gibco) supplemented with 10 % FBS, 100 units/ml penicillin, 100 µg/ml streptomycin, 250 μg/ml amphotericin B and 5 μg/ml plasmocin, at 37 °C in a humidified atmosphere of 5 % (v/v) CO_2_/air.

### 3-(4,5-dimethylthiazol-2-yl)-2,5-diphenyltetrazolium bromide (MTT) assay

 SH-SY5Y cells were washed with PBS, detached by mechanical “flushing” and seeded in D-MEM without phenol red and FBS at a density of 200 000 cells/cm^2^ in 96-well plates and grown over night. SOD1 protein was incubated with the cells for 72 h, and cell viability was assessed at the end of the experiment by addition of MTT in a final concentration of 5 mg/ml and further incubation for 4 h. The formazan product was dissolved with 20 % SDS in 50 % dimethyl formamide, and absorbance measured at 570 nm (Spectramax 340 PC). Absorbance values were normalized against the buffer control set to 100 % viability. To have a measure of the total viability response in Figure 1E, the area under each viability curve was calculated using Graphpad prism 5.0 and normalized against buffer.

### SDS-PAGE

 Samples collected from the cell culture were centrifuged at 13 300 x g for 10 min and mixed with 2x sample buffer (4 % SDS, 125 mM Tris, pH 6.8) with or without 1/10 volume beta-mercaptoethanol. To the sample buffer without beta-mercaptoethanol, 100 mM of iodoacetamide was added to avoid disulfide scrambling/crosslinking during heat treatment and electrophoresis. The samples were placed in nearly boiling water for 5 min and separated on a 14 % Tris-glycine polyacrylamide gel (Invitrogen), or alternatively an anykD gel (Biorad) used in Figure 3. The gel was run at 125 V in cold room, and stained with coomassie blue.

### Thioflavin T aggregation assay

 Apo treated SOD1 samples with a protein concentration of 100 μM were mixed with 1/10 volume of 100 μM ThT and loaded on a non-treated 96 well polystyrene plate (Nunc). The plate was sealed with a foil plate seal and incubated at 37°C without agitation. ThT fluorescence was measured in a FLUOStar Omega plate reader using an excitation wavelength of 444 nm and an emission wavelength of 485 nm. All aggregation experiments were performed in PBS pH 7.4 (50 mM phosphate 100 mM NaCl).

### Electron Microscopy

 Samples collected from the aggregation plate at the end-point of the experiment were diluted in H_2_O to 25 μM. Carbon-coated 200-mesh copper grids were applied to a drop of the protein solution and blotted for 5 min. The grids were subsequently applied to a drop of water, briefly dried, and stained with 1% (w/v) uranyl acetate or 1 % (w/v) phosphotungstic acid. Images were recorded in a Tecnai G^2^ Spirit BioTWIN microscope with a tungsten filament operating at 80 kV. Measurements of oligomer dimensions were performed with Image J.

### CD spectroscopy

 CD spectra of apoSOD1 were acquired using a Jasco J-815 equipped with a peltier temperature controller. The CD signal (θ) was integrated between 222 and 236 nm. The melting temperature (*T*_m_) was determined by plotting and fitting θ vs. T as described in [9]. 

## Supporting Information

Figure S1 **Iodoacetamide treatment of wild-type holoSOD1 alkylates a single cysteine.** “As it comes” wild-type holoSOD1 was alkylated with iodoacetamide and analysed with MALDI-TOF. (**A**) Mass spectra of dimeric wild-type (IAM) with mainly one peak of 15860.5 Da representing SOD1 with one alkylated cysteine. (**B**) Mass spectra of monomeric (F50E/G51E) wild-type (IAM) with mainly one peak of 15914 Da representing SOD1 with one alkylated cysteine.
(TIF)

Figure S2 **SDS-PAGE of apoSOD1.** Apo-treated SOD1 proteins were separated on a 14 % tris-glycine polyacrylamide gel under (**A**) non-reducing and (**B**) reducing conditions. All protein variants migrate mainly with the same rate as apoSOD1 C6/111A under non-reducing conditions, indicative of a protein with the native disulfide intact.
(TIF)

Figure S3 **Melting curves of monomeric (F50E/G51E) and dimeric apoSOD1 wild-type, apoSOD1 wild-type (IAM) and apoSOD1 C6/111A.** SOD1 was apo-treated according to experimental procedures, and further diluted in phosphate buffer, pH 7.5. Thermal denaturation was accomplished by increasing the temperature (T) from 5 to 95°C. For each temperature increase, the CD signal was integrated between 222 and 236 nm and plotted as a function of (T). Melting temperatures (Tm) were determined by fitting the data as described in [9] using graphpad prism v. 5.0. All proteins display normal melting curves with distinct transitions, indicative of a native protein.
(TIF)

Figure S4 **SDS-PAGE of apoSOD1 proteins collected from the cell culture.** ApoSOD1 proteins collected from the cell culture were separated on a 14 % tris-glycine polyacrylamide gel under (**A**) non-reducing and (**B**) reducing conditions. Monomeric (F50E/G51E) and dimeric apoSOD1 wild-type, wild-type (IAM) and C111A migrate with a reduced rate, indicative of a disulfide scrambled protein. High molecular weight aggregates are also observed, but with lower intensity. Virtually no staining is seen with cell media from wells without SOD1 added, either with (1) dead cells (killed by H_2_O_2_) or (2) viable cells (buffer ctrl).
(TIF)

Figure S5 **Individual aggregation curves in PBS.** ApoSOD1 (90 μM) was aggregated with 10 μM ThT in PBS without agitation for 83h (4950 min). Data was collected from three separate experiments run in duplicate and plotted with 50 min interval. All dimeric proteins display only weak ThT fluorescence under these conditions, whereas monomeric (on a F50E/G51E background) C111A, wild-type and wild-type (IAM) display high ThT signals. The spread of the data from different experiments is generally high for aggregating proteins, especially monomeric C111A and wild-type (IAM). The experimental variation between duplicate samples within the same experiment is low.
(TIF)

Figure S6 **Aggregates formed from monomeric apoSOD1 C111A stained with phosphotungstic acid (PTA).** Aggregated monomeric apoSOD1 C111A (on a F50E/G51E background) was applied to a carbon-coated copper grid and stained with PTA. The aggregates formed are indistinguishable from the aggregates stained with uranyl acetate (Figure 3C).
(TIF)
