# Supplementary figures and images for: Disulfide Scrambling in Superoxide Dismutase 1 Reduces Its Cytotoxic Effect in Cultured Cells and Promotes Protein Aggregation
Source: PLoS One. 2013 Oct 15;8(10):e78060. doi: 10.1371/journal.pone.0078060 (PMC3797058; doi:10.1371/journal.pone.0078060)

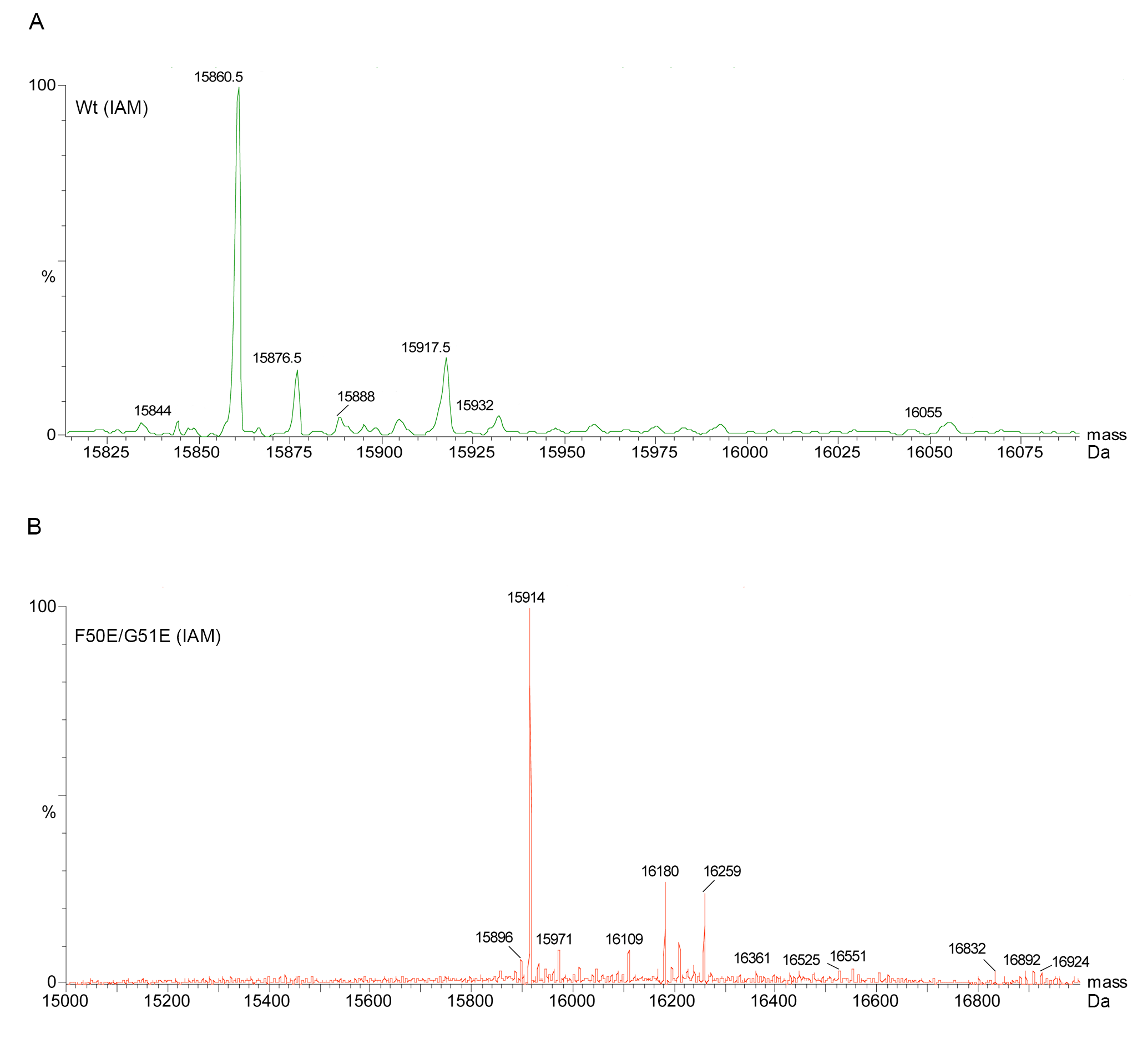

Supplement: Figure S1 — Iodoacetamide treatment of wild-type holoSOD1 alkylates a single cysteine. “As it comes” wild-type holoSOD1 was alkylated with iodoacetamide and analysed with MALDI-TOF. (A) Mass spectra of dimeric wild-type (IAM) with mainly one peak of 15860.5 Da representing SOD1 with one alkylated cysteine. (B) Mass spectra of monomeric (F50E/G51E) wild-type (IAM) with mainly one peak of 15914 Da representing SOD1 with one alkylated cysteine. (TIF) [file pone.0078060.s001.tif]

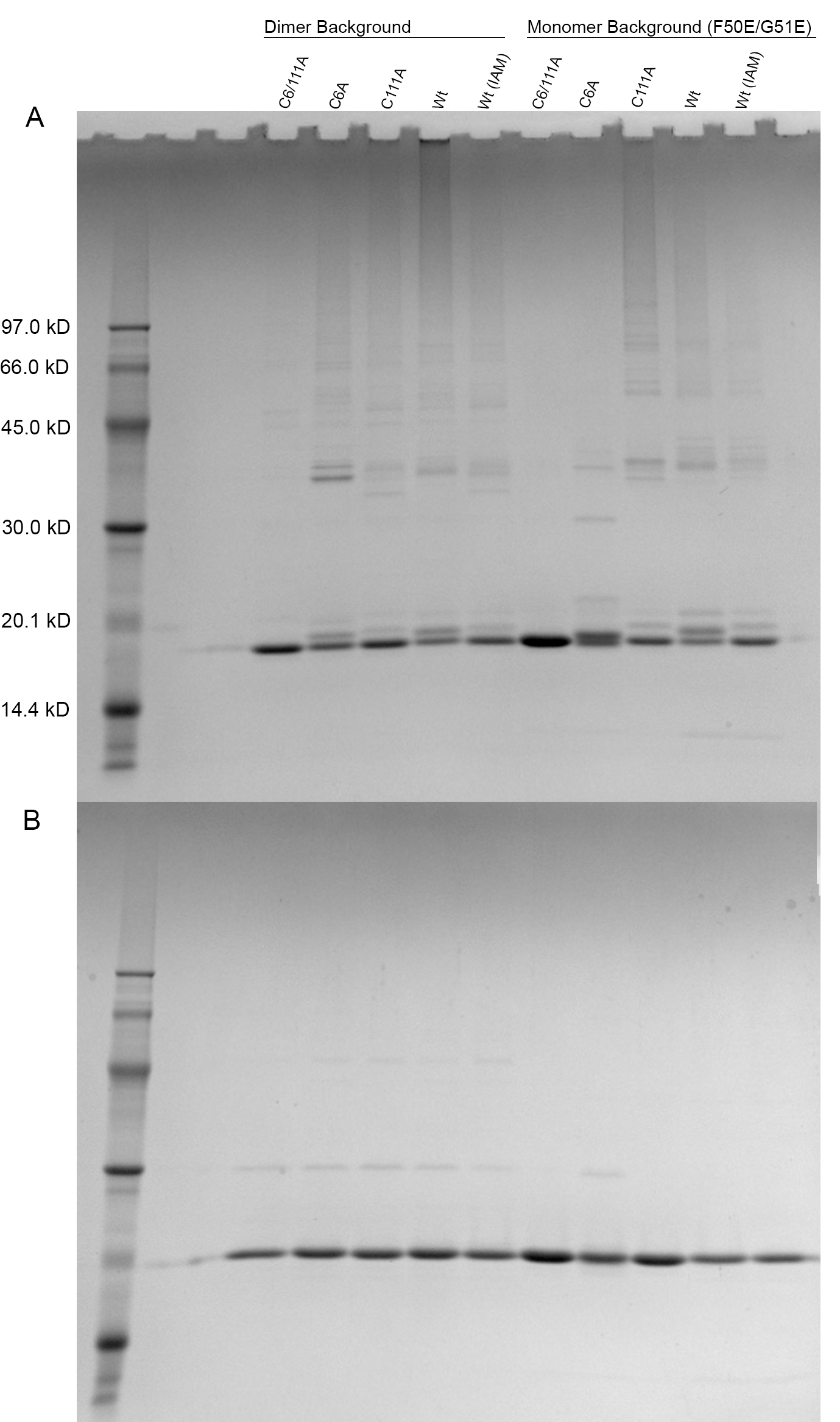

Supplement: Figure S2 — SDS-PAGE of apoSOD1. Apo-treated SOD1 proteins were separated on a 14 % tris-glycine polyacrylamide gel under (A) non-reducing and (B) reducing conditions. All protein variants migrate mainly with the same rate as apoSOD1 C6/111A under non-reducing conditions, indicative of a protein with the native disulfide intact. (TIF) [file pone.0078060.s002.tif]

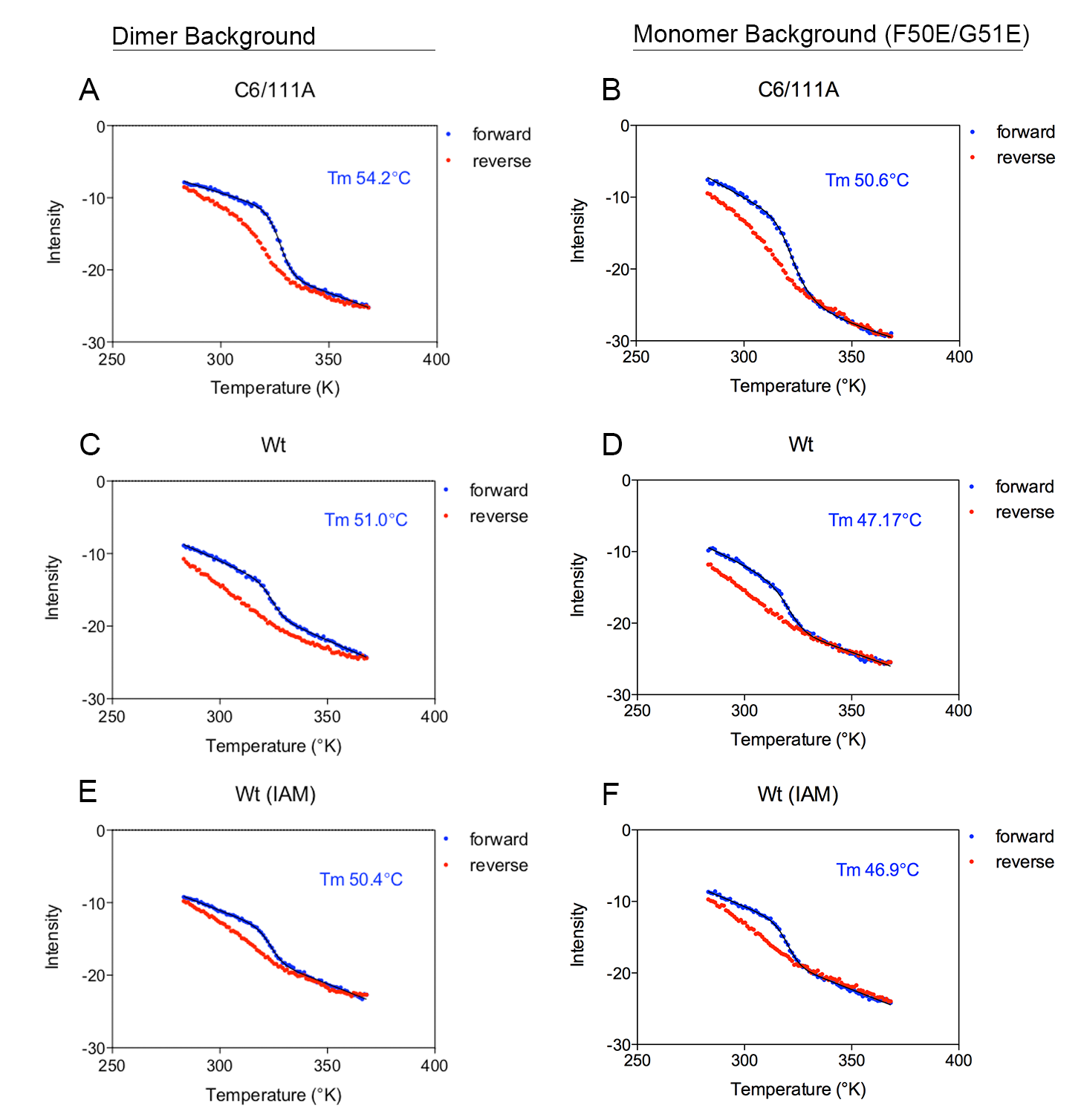

Supplement: Figure S3 — Melting curves of monomeric (F50E/G51E) and dimeric apoSOD1 wild-type, apoSOD1 wild-type (IAM) and apoSOD1 C6/111A. SOD1 was apo-treated according to experimental procedures, and further diluted in phosphate buffer, pH 7.5. Thermal denaturation was accomplished by increasing the temperature (T) from 5 to 95°C. For each temperature increase, the CD signal was integrated between 222 and 236 nm and plotted as a function of (T). Melting temperatures (Tm) were determined by fitting the data as described in [9] using graphpad prism v. 5.0. All proteins display normal melting curves with distinct transitions, indicative of a native protein. (TIF) [file pone.0078060.s003.tif]

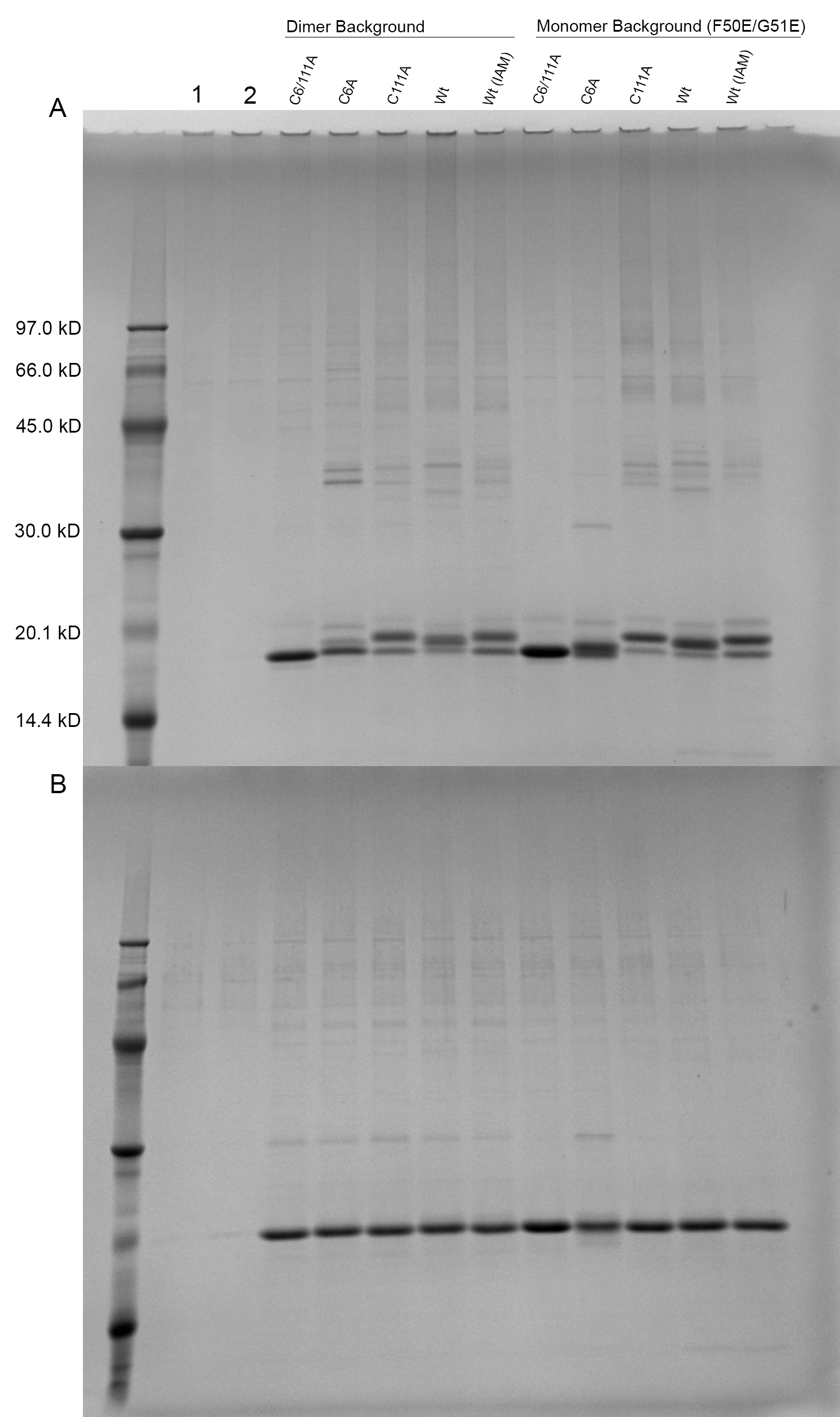

Supplement: Figure S4 — SDS-PAGE of apoSOD1 proteins collected from the cell culture. ApoSOD1 proteins collected from the cell culture were separated on a 14 % tris-glycine polyacrylamide gel under (A) non-reducing and (B) reducing conditions. Monomeric (F50E/G51E) and dimeric apoSOD1 wild-type, wild-type (IAM) and C111A migrate with a reduced rate, indicative of a disulfide scrambled protein. High molecular weight aggregates are also observed, but with lower intensity. Virtually no staining is seen with cell media from wells without SOD1 added, either with (1) dead cells (killed by H2O2) or (2) viable cells (buffer ctrl). (TIF) [file pone.0078060.s004.tif]

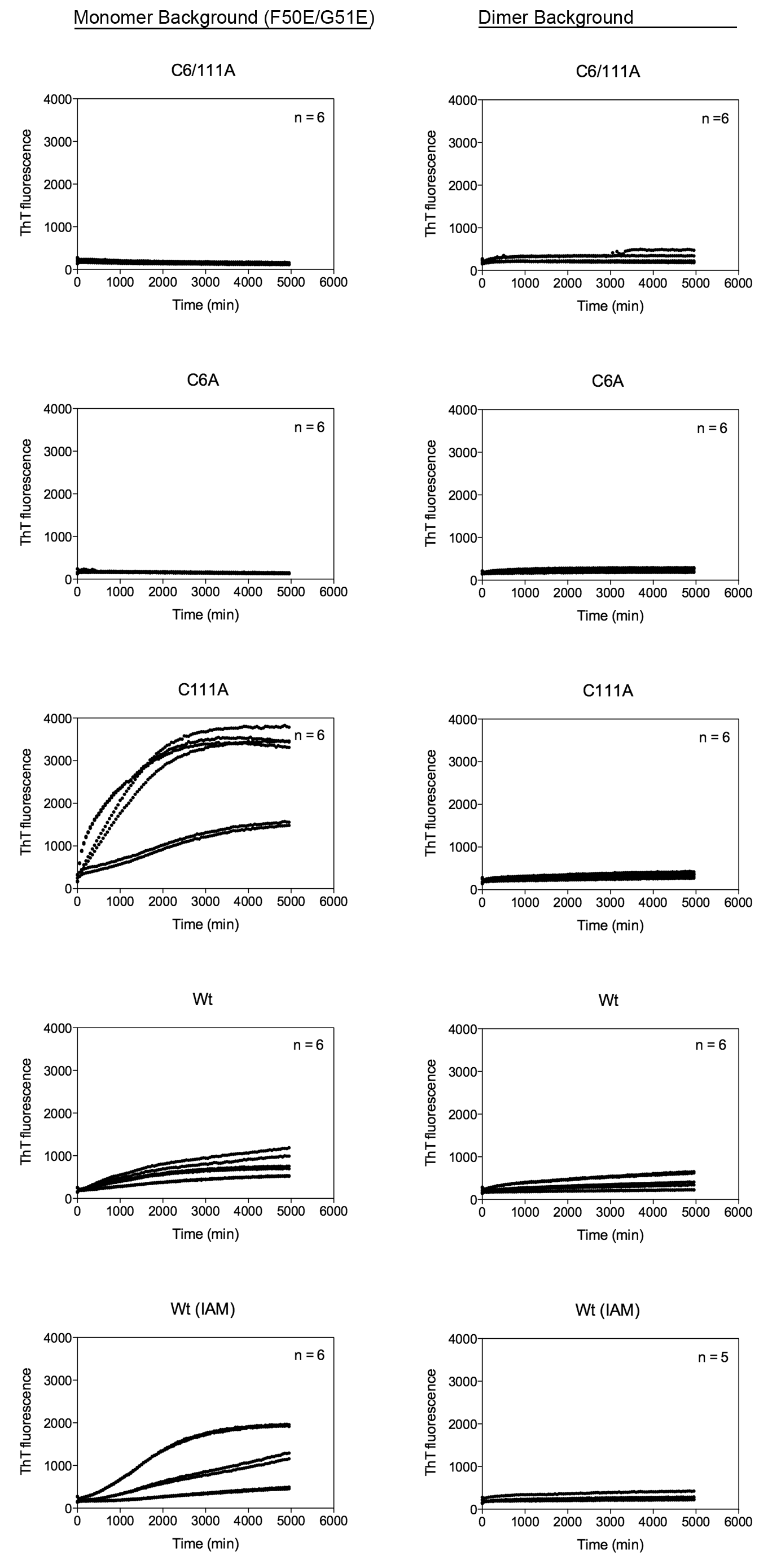

Supplement: Figure S5 — Individual aggregation curves in PBS. ApoSOD1 (90 μM) was aggregated with 10 μM ThT in PBS without agitation for 83h (4950 min). Data was collected from three separate experiments run in duplicate and plotted with 50 min interval. All dimeric proteins display only weak ThT fluorescence under these conditions, whereas monomeric (on a F50E/G51E background) C111A, wild-type and wild-type (IAM) display high ThT signals. The spread of the data from different experiments is generally high for aggregating proteins, especially monomeric C111A and wild-type (IAM). The experimental variation between duplicate samples within the same experiment is low. (TIF) [file pone.0078060.s005.tif]

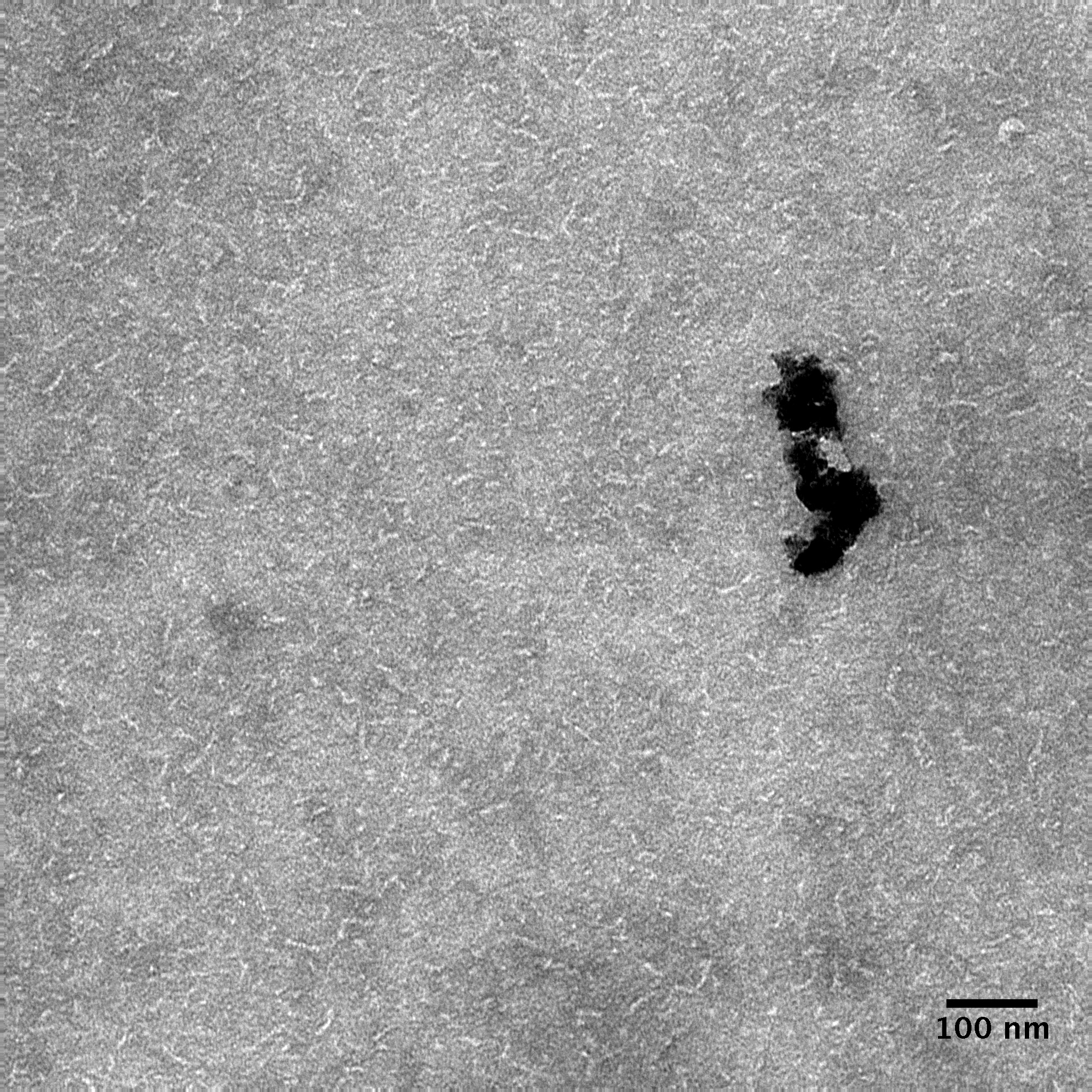

Supplement: Figure S6 — Aggregates formed from monomeric apoSOD1 C111A stained with phosphotungstic acid (PTA). Aggregated monomeric apoSOD1 C111A (on a F50E/G51E background) was applied to a carbon-coated copper grid and stained with PTA. The aggregates formed are indistinguishable from the aggregates stained with uranyl acetate (Figure 3C). (TIF) [file pone.0078060.s006.tif]
